# Low relationship quality predicts scratch contagion during tense situations in orangutans (*Pongo pygmaeus*)

**DOI:** 10.1002/ajp.23138

**Published:** 2020-04-24

**Authors:** Daan W. Laméris, Evy van Berlo, Elisabeth H. M. Sterck, Thomas Bionda, Mariska E. Kret

**Affiliations:** ^1^ Institute of Psychology, Faculty of Social Sciences, Cognitive Psychology Unit Leiden University Leiden The Netherlands; ^2^ Department of Biology, Animal Ecology Research Group Utrecht University Utrecht The Netherlands; ^3^ Leiden Institute for Brain and Cognition Leiden The Netherlands; ^4^ Department of Animal Science Biomedical Primate Research Centre Rijswijk The Netherlands; ^5^ Apenheul Primate Park Apeldoorn The Netherlands

**Keywords:** arousal, behavioral contagion, great ape, orangutan, scratching

## Abstract

Primates show various forms of behavioral contagion that are stronger between kin and friends. As a result, behavioral contagion is thought to promote group coordination, social cohesion, and possibly state matching. Aside from contagious yawning, little is known about the contagious effect of other behaviors. Scratching is commonly observed during arousal and as such may play a role within group dynamics. While the Bornean orangutan (*Pongo pygmaeus*) is commonly considered the least social great ape, orangutans do engage in social interactions. Therefore, their social organization makes them a suitable case for studying the social function of behavioral contagion. Through behavioral observations of captive orangutans, we recorded all yawn and scratch events together with the corresponding behavior of all bystander group‐members. As yawning was rarely observed, no conclusions could be drawn regarding this behavior. Scratching was contagious and occurred within 90 s after the triggering scratch. Specifically, orangutans showed increased scratch contagion when they had seen a weakly bonded individual scratch during tense contexts. When the orangutan had not seen the triggering scratch, the contagiousness of scratching was not affected by context or relationship quality. Our results indicate that behavioral contagion is not simply higher between individuals with stronger social relationships, but that the contagiousness of behaviors may vary based on the context and on social factors. We discuss these findings in light of an adaptive function that may reduce aggression.

## Research Highlights

- Bornean orangutans show scratch contagion.
- Seen scratches are more contagious during tense context between nonkin/friends.

## Graphical abstract

Scratch contagion is stronger between weakly bonded individuals (nonkin/friends) during tense situations.

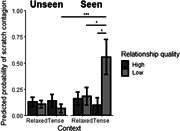

## 1 INTRODUCTION

Behavioral contagion is a phenomenon in which a behavior is automatically triggered by the perception of a similar behavior in others (Zentall, 2003). From a proximate perspective, such contagion can be explained by mechanisms rooted in primitive forms of state matching and empathetic processing (Joly‐Mascheroni, Senju, & Shepherd, 2008; Palagi, Leone, Mancini, & Ferrari, 2009). The perception‐action mechanism explains that if such behaviors are manifestations of emotions, contagion can result in emotional state‐matching, a phenomenon known as emotional contagion (Preston & de Waal, 2002b). However, behavioral contagion can also be explained more parsimoniously as the nonconscious mimicry of a partner's behavior (Massen & Gallup, 2017; Yoon & Tennie, 2010). Interestingly, forms of behavioral contagion are commonly found to be stronger between kin and friends (Campbell & de Waal, 2011; Demuru & Palagi, 2012; Massen, Vermunt, & Sterck, 2012; Palagi et al., 2009; Palagi, Norscia, & Demuru, 2014). Such enhanced behavioral contagion between individuals that share social connections is thought to facilitate group coordination and social cohesion (Lakin, Jefferis, Cheng, & Chartrand, 2003; Clay & de Waal, 2013; Preston & de Waal, 2002a; Prochazkova & Kret, 2017).

Probably the most well‐studied behavior within the behavioral contagion literature is yawning. While spontaneous yawning (i.e., nonsocial yawning) is widespread across vertebrates and may function in promoting cortical arousal (Baenninger, 1997; Guggisberg, Mathis, Schnider, & Hess, 2010; Vick & Paukner, 2010), and/or changing emotional states through decreasing brain temperature (Gallup & Gallup, 2008; Massen, Dusch, Eldakar, & Gallup, 2014; Massen & Gallup, 2017), contagious yawning is restricted to fewer species in which this trait may have evolved independently (Massen & Gallup, 2017).

Thus far, contagious yawning is observed in several primate species, including chimpanzees (*Pan troglodytes*; Anderson, Myowa‐Yamakoshi, & Matsuzawa, 2004; Campbell & de Waal, 2011; Campbell, Carter, Proctor, Eisenberg, & de Waal, 2009; Massen et al., 2012), bonobos (*P. paniscus*; Demuru & Palagi, 2012; Palagi et al., 2014), and gelada baboons (*Theropithecus gelada*; Palagi et al., 2009). Other species in which contagious yawning is observed include domesticated dogs (*Canis lupus familiaris*; Joly‐Mascheroni et al., 2008; Madsen & Persson, 2013), wolves (*C. lupus lupus*; Romero, Ito, Saito, & Hasegawa, 2014; Romero, Konno, & Hasegawa, 2013), budgerigars (*Melopsittacus undulates*; Gallup, Militello, Swartwood, & Sackett, 2017; Miller, Gallup, Vogel, Vicario, & Clark, 2012), and elephant seals (*Mirounga leonina*; Wojczulanis‐Jakubas, Plenzler, & Jakubas, 2018). However, some experimental studies have failed to provide convincing evidence for yawn contagion in bonobos, orangutans (*Pongo abelli*), and gorillas (*Gorilla gorilla*; Amici, Aureli, & Call, 2014), stump‐tailed macaques (*Macaca arctoides*; Paukner & Anderson, 2006), ring‐tailed lemurs (*Lemur catta*), and ruffed lemurs (*Varecia variegata*; Reddy, Krupenye, MacLean, & Hare, 2016), dogs (Harr, Gilbert, & Phillips, 2009), and red‐footed tortoises (*Geochelone carbonaria*; Wilkinson, Sebanz, Mandl, & Huber, 2011). This illustrates the ongoing debate on the possible mechanism underlying contagious yawning.

Although not receiving as much attention as contagious yawning, scratching may be another interesting behavior for contagion studies. Scratching is commonly associated with the presence of psychological and physiological stress (Maestripieri, Schino, Aureli, & Troisi, 1992; Schino, Troisi, Perretta, & Monaco, 1991; Troisi, 1999). For example, increased scratch rates have been reported during aggressive interactions (Palagi & Norscia, 2011), postconflict interactions without reconciliation (reviewed in Aureli, Cords, & Van Schaik, 2002), dominance‐related interactions (Kaburu, MacLarnon, Majolo, Qarro, & Semple, 2012; Peignot, Jankowski, & Anderson, 2004), and predation attempts (Palagi & Norscia, 2011). Concurrently, scratching behavior is reduced after play bouts (Norscia & Palagi, 2011), during affiliative interactions (Aureli & Yates, 2010), and after reconciliation following aggressive interactions (Aureli, Van Schaik, & Van Hooff, 1989). However, a recent study also found that scratching increases with positive arousal (e.g., during play bouts), suggesting that scratching may be a marker of general emotional arousal, rather than an indicator of negative emotions specifically (Neal & Caine, 2016).

Apart from benefits for the expresser (Koolhaas et al., 1999), scratching potentially signals arousal to other group‐members (Bradshaw, 1993). In rhesus macaques (*Macaca mulatta*), for example, scratching reduces the likelihood of subsequent aggression and increases the chance of affiliative interactions (Whitehouse, Micheletta, & Waller, 2017). Furthermore, stressed individuals are a potential threat to group‐members as they tend to behave unpredictably (Aureli, Cozzolinot, & Cordischif, 1992). As such, the recognition and acquisition of the emotions of aroused individuals can result in fewer costly interactions (Whitehouse, Micheletta, Kaminski, & Waller, 2016). While these studies suggest that scratching may play an important role within social groups, the contagious effect of scratching and its potential function is poorly understood.

Most studies on behavioral contagion in great apes focused on bonobos and chimpanzees, probably because of their complex social structures, advanced cognitive capacities, and evolutionary proximity to humans (MacLean, 2016). However, the orangutan too, is one of our closest living relatives with highly developed cognitive skills (Damerius et al., 2019; Van Schaik et al., 2003), yet is considered semi‐solitary as it does not live in stable social groups (Delgado & Van Schaik, 2000; Galdikas, 1985; Mitra Setia, Delgado, Utami Atmoko, Singleton, & van Schaik, 2009; Singleton, Knott, Morrogh‐Bernard, Wich, & van Schaik, 2009; Van Schaik, 1999). Nonetheless, orangutans still form temporary parties for social reasons, e.g. for mating opportunities, protection from male coercion, and socialization opportunities for infants (Mitani, Grether, Rodman, & Priatna, 1991; Mitra Setia et al., 2009; Singleton et al., 2009; Van Schaik, 1999). Furthermore, zoo‐housed orangutans show increased frequencies of social behavior, including agonistic interactions (Edwards & Snowdon, 1980; Tajima & Kurotori, 2010; Zucker, 1987). This suggests that orangutans show a certain degree of behavioral flexibility under social contexts which makes them an interesting case for a study on behavioral contagion and its possible social function.

Research on behavioral contagion in orangutans, however, is scarce. One study found that orangutans show rapid facial mimicry during play events (Davila Ross, Menzler, & Zimmermann, 2008), while another study did not find evidence of yawn contagion in an experimental setup (Amici et al., 2014). In this study, we aimed to enhance our understanding of the function of behavioral contagion in the orangutan. To do so, we focused on yawning as this behavior is commonly studied in behavioral contagion research. In addition, we decided to focus on scratching behavior because of its possible link to arousal (Elder & Menzel, 2001). As such, we recorded all yawning and scratching events in a group of zoo‐housed Bornean orangutans (*P. pygmaeus*) with the aim to investigate whether (a) yawning and scratching is contagious and (b) whether contagion has a social function in this species. Based on a previous study reporting the presence of rapid facial mimicry (Davila Ross et al., 2008), we hypothesize that behavioral contagion is present and extends to yawning and scratching behavior. Furthermore, if these behaviors have a social function, we expect that the contagion of yawning and scratching will be influenced by the relationship quality of the expresser and observer and that contagion is higher between kin and friends.

## 2 METHODS

### 2.1 Ethics

The care and housing of the orangutans was adherent to the guidelines of the EAZA Ex situ Program. Only observational data were collected, therefore there was no need for the approval of the Ethics Committee of Apenheul. The study complied with the requirements of the Dutch Animal Care and Use Committee and conformed to the American Society of Primatologists Principles for the Ethical Treatment of Non‐Human Primates.

### 2.2 Study subjects and data collection

Behavioral data were collected from February to May 2017 on nine adult Bornean orangutans (three males and six females, mean age= 23.2, range= 7–52 years old, see Table S1) housed in Apenheul Primate Park, The Netherlands. The animals were housed in a building consisting of four indoor enclosures that were each connected to outdoor islands. The four enclosures could be disconnected from and connected to two adjacent enclosures, which allowed the zookeepers to alter group composition on a daily basis, based on the animals' preferences. Usually, there were four separate groups (ranging from one to four individuals) that differed in composition and occasionally three groups (ranging from two to five individuals). This housing environment aims to mimic the natural social structure of orangutans in which they form temporary parties but no stable social groups. Some individuals were never housed together to avoid conflict (e.g., the two adult males). Focal‐animal sampling of 10 min sessions was used to score behavioral patterns including social behaviors (e.g., grooming, agonistic interactions, and sexual behaviors), locomotion (e.g., walking and climbing), and food‐associated behavior (e.g., foraging and feeding; ~18.5 hr per focal; Table S1, and see Table S2 for the ethogram). We used all‐occurrence sampling to record all yawning and scratching events of group‐members in the subgroup of the focal animal for 165 hr in total (Altmann, 1974). Observations were performed by one trained researcher from the visitor's area in both indoor and outdoor enclosures. Due to the relatively low temperatures during the observation period, the orangutans were kept inside and as such most observations were performed in the indoor enclosures. The indoor enclosures were ~60 m^2^ in which observation conditions were excellent; the researcher had full view of the enclosure and its individuals as there were no big constructions blocking the line of sight. In addition, because subgroups had a maximum of five individuals, and because yawning and scratching could be considered “attention‐attracting” behaviors (Demuru & Palagi, 2012), it was possible for the researcher to record all yawning and scratching events. The following variables were recorded whenever a yawn or scratch occurred: (a) time of occurrence; (b) identity of the expresser; (c) identity of all possible observers (i.e., individuals that were within the same enclosure); (d) presence/absence of a contagious response (i.e., a congruent behavior) within 3 min following the last triggering event (i.e., a spontaneous yawn or scratch); (e) time latency in contagious response measured in seconds (s); (f) duration of scratching behavior (short; <5 s or long; > 5 s); (g) if the observer could see the triggering event or not, based on the facial direction of the observer; (h) estimated distance between the expresser and observer (<1 m, 1–5 m, 5–10 m, and >10 m); and (i) the context in which the triggering event occurred, categorized as “tense” or “relaxed.” The context categorization was based on the behavior of the expresser before and after the yawning or scratching behavior. Behaviors that indicated tension included display behavior (e.g., charging and shaking of climbing structures), high arousal vocalizations (long‐calls or kiss squeaks), or agonistic behaviors (direct aggression and chasing). Because we rarely observed agonistic interactions, we consider yawning and scratching to be related to levels of increased arousal, but not aggression. Relaxed contexts were characterized by behaviors such as foraging, resting, or socio‐positive interactions (e.g., grooming). To ensure the reliability of our data, we restricted our data set to the indoor observations and excluded cases for which the expresser and observer were at a greater distance than 10 m.

### 2.3 Relationship quality

Scan‐sampling was performed every 30 min to score allogrooming, contact sitting, social play, and sexual behaviors (e.g., mounting and genital contact) to calculate a relationship quality with a corrected composite sociality index (CSI; Silk, Altmann, & Alberts, 2006). Relationship quality was based on two levels: kinship and CSI (Demuru & Palagi, 2012; Palagi et al., 2014). Regarding kinship, only maternal lineages were considered (*r* = .5), resulting in four dyads. However, the dyad involving a juvenile male was excluded from the analyses and only three kin dyads remained. One of these dyads was a mother that, in the past, already had an offspring and took on the role of surrogate mother for another juvenile of the same age as her own. CSI was calculated to identify high and low relationship qualities (Silk et al., 2006). The CSI is a useful measure for scoring how much the positive relationship of a particular dyad deviates from the average of all dyads. Since group composition for the orangutans was regularly changed and based on the preferences of the orangutans, we corrected for the total number of days spent together per dyad. Dyads with CSI scores in the top quartile were considered to have a high relationship quality, *N* = 5 (Demuru & Palagi, 2012), which included the kin dyads. Because of the low number of kin dyads, we did not separately test the influence of kinship on the degree of contagion. All other dyads were considered to have a low relationship quality, *N* = 9.

### 2.4 Statistics

Yawn and scratch rates were extracted for two conditions: the baseline condition and the contagious condition. The baseline condition included spontaneous yawn and scratch events (i.e., when subgroup‐members did not show yawning or scratching) which were extracted from the focal‐animal observations. The contagious condition included those yawn/scratch events that occurred within a 3‐min period after a congruent triggering behavior, hence after spontaneous yawning/scratching behavior. By means of all‐occurrence sampling, a total of 95 yawn and 597 scratch events were recorded. We had insufficient data to statistically analyze yawn contagion (baseline *N* = 52 and contagion *N* = 4) and therefore focused on the contagiousness of scratching.

To test the data for normality, the Shapiro–Wilk test was used and Levene's test for equality of variances was used to test for homoscedasticity. The use of long timeframes to study contagious responses have been discussed (Massen & Gallup, 2017). For this reason, we investigated the temporal boundaries of scratch contagion (i.e., during which time period following a triggering scratch of a group‐member were scratch rates higher as compared to scratch rates observed during baseline). As such, we divided the scratch rates during the 3 min contagious condition into six intervals of each 30 s and calculated individual contagious scratch rates for each of the six 30 s intervals. In addition, for each individual, we calculated one baseline scratch rate per 30 s (i.e., number of spontaneous scratches per 30 s, derived from the focal sampling data). Due to the small sample size, we used bootstrapped paired samples *t* tests to compare each 30 s interval in the contagious condition to their matched 30 s baseline scratch rate. We employed Bonferroni corrections to adjust for multiple comparisons with the 30 s baseline scratch rate. From this, we found that contagious scratch rates were only higher than baseline scratch rates during the first three intervals (i.e., the first 90 s after a triggering scratch; Figure S1). Therefore, we only considered those scratches happening within 90 s after a triggering scratch as contagious and excluded the scratches that occurred after 90 s (*n* = 37). We then pooled the contagious scratches that occurred within 90 s together and calculated individual scratch rates during this period. We also calculated a baseline scratch rate per 90 s and compared this to the contagious scratch rates using a bootstrapped paired samples *t* test.

We created a generalized linear mixed model (GLMM) that included the identity of the expresser and observer as random effect and “context” (categorical; tense vs. relaxed), “relationship quality” (categorical; high versus low relationship quality) as fixed factors to test their effect on the occurrence of scratch contagion. Furthermore, we decided to include “seeing the triggering scratch” (categorical; seen vs. unseen) as additional fixed factor since auditory cues of scratching can already be sufficient to induce a contagious response in humans (Swithenbank, Cowdell, & Holle, 2016). We included a three‐way interaction for context, relationship quality and seeing the triggering scratch because we hypothesized that contagious responses triggered by unseen scratches would not be influenced by relationship quality, simply because the observer did not have information about the expresser. Sex of the expresser, observer, and sex combination were considered as additional fixed factors, but due to the low sample sizes (three males and six females), we decided to leave them out. The models used a binomial distribution (contagion or no contagion) and a logit link function. Likelihood ratio tests and a *χ* ^2^ distribution were used to compare the full model with the null model. Multicollinearity between independent variables was tested and variables with a variance inflation factor (VIF) of > 5 were rejected from the model (O'Brien, 2007). None of the factors showed high VIF values. Analyses were conducted using R version 3.6.1 (R Core Team, 2016), with the GLMM calculated using the lme4 package (Bates, Maechler, & Bolker, 2014).

## 3 RESULTS

### 3.1 Orangutans are susceptible to scratch contagion

**Figure 1:**
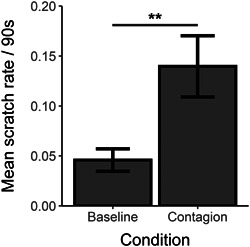
Mean scratch rates (±*SEM*) per 90 s in the baseline and contagion condition. *SEM*, standard error of mean. **p < .01

We compared the scratch rates during the baseline condition with the scratch rates in each of the 30 s intervals during the contagious condition. Orangutans scratched more during the first 90 s after a triggering scratch (Figure S1; bootstrapped paired samples *t* test: Baseline vs. 0–30 s: *p* < .001; Baseline vs. 31–60 s: *p* < .001; Baseline vs. 61–90: *p* = .002). Furthermore, the scratch rates over the 90 s contagious condition were higher than the 90 s baseline condition (Figure 1; bootstrapped paired samples *t* test: *p* < .001). This suggests that only those scratches happening within 90 s after another scratch can be considered contagious.

### 3.2 Factors influencing scratch contagion

**Figure 2:**
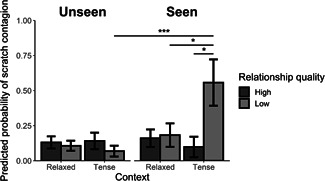
Predicted probability of scratch contagion (±*SEM*) based on the three‐way interaction between seeing the triggering scratch, context and relationship quality. *SEM*, standard error of mean. *p < .05; ***p < .001

We further assessed potential factors explaining the occurrence of scratch contagion. Overall, the full model fitted the data better than the null model, as the likelihood ratio test (LRT) revealed a significant effect of the predictors on the occurrence of contagious scratching (LRT: $\chi_{7}^{2}$= 16.291, *p* = .023). We found a significant interaction between whether the triggering scratch was seen or not, context and relationship quality (Table 1). Specifically, we found no difference in scratch contagion between context and relationship quality when the observer had not seen the triggering scratch. However, using simple contrasts, we found that during tense contexts, scratch contagion is more likely to occur between individuals that share a low relationship quality when the observer had seen the triggering scratch compared with when the observer had not seen the scratch (Figure 2; *z* = 3.616, *p* < .001). Furthermore, when only considering the cases where the observer had seen the triggering scratch, we found that scratching is more contagious between individuals that shared a low relationship quality during tense contexts compared with relaxed contexts (*z* = 2.301, *p* = .021) and during tense context between individuals that shared a low relationship quality compared with a high relationship quality (*z* = 2.348, *p* = .019). Follow‐up analyses suggest that this effect is not a by‐product of increased visual attention towards individuals with a low relationship quality as more scratches were observed when the expresser and observer shared a high relationship quality ($\chi_{1}^{2}$= 17.871, *p* < .001).

**Table 1:** Type III tests for fixed effects on the occurrence of scratch contagion

	Estimate	*SE*	$\chi_{1}^{2}$	*p*
Intercept	−1.897	0.380	24.864	*<.001*
Context (tense)	0.088	0.457	0.038	.846
Relationship quality (low)	−0.228	0.428	0.283	.595
Seen/unseen (seen)	0.240	0.418	0.330	.566
Context × relationship quality (tense × low)	−0.576	0.725	0.631	.427
Context × seen/unseen (tense × seen)	−0.653	0.956	0.466	.495
Relationship quality × seen/unseen (low × seen)	0.384	0.675	0.324	.569
*Context* × *relationship quality* × *seen/unseen (tense* × *low* × *seen)*	2.869	1.334	4.627	*.032*

*Note:* GLMMs were used with a binomial distribution and logit link function. Effects with *p* < .05 are depicted in italics.

Abbreviations: GLMMs, generalized linear mixed models; *SE*, standard error.

It is possible that the increased scratch rates during tense context do not reflect contagion, but are simply a by‐product of increased arousal levels during tense contexts in general (Castles & Whiten, 1998). Follow‐up analyses revealed that contagious scratch rates did not differ between tense and relaxed context (bootstrapped paired samples *t* test: *p* = .795), suggesting that the observed effect of context is not just a by‐product of increased scratching due to increased stress levels during tension.

## 4 DISCUSSION

The contagion of behaviors such as yawning and scratching and their possible social function remain poorly understood. The current study aimed to investigate whether yawning and scratching are contagious in the orangutan and whether the contagion of these behaviors is linked to the context in which these behaviours occur and the quality of the bond between individuals. Orangutans showed increased scratch rates after a group‐member scratched, indicating behavioral contagion. This effect was visible within the first 90 s after the triggering scratch. Furthermore, when the relationship quality between the expresser and observer was low, and the observer had seen the triggering scratch, scratch contagion was more likely to occur during tense situations.

Our observation that scratch contagion is stronger in a tense context between weakly bonded individuals is novel, as most other studies report increased behavioral contagion between individuals with a high relationship quality (Campbell & de Waal, 2011; Demuru & Palagi, 2012; Massen et al., 2012; Palagi, Leone et al., 2009; Palagi, Norscia et al., 2014). Yet, these studies predominantly looked at yawn contagion for which the social function and emotional load is debated and for which it is unknown how others perceive this behavior (Gallup, 2011; Massen & Gallup, 2017; Palagi, Celeghin, Tamietto, Winkielman, & Norscia, 2020). Scratching, on the other hand, is often associated with physiological and psychological stress (Maestripieri et al., 1992; Schino et al., 1996; Troisi et al., 1991) although there is growing evidence that scratching also increases during positive arousing events, such as during play bouts (Neal & Caine, 2016). Without further measures (e.g., changes in emotional valence with cognitive bias testing as done by Adriaense, Martin, Schiestl, Lamm, & Bugnyar, 2019 and Saito, Yuki, Seki, Kagawa, & Okanoya, 2016), we cannot conclude which emotions underlie scratching and if scratch contagion is truly a form of emotional contagion. Nonetheless, emotional contagion consists of simpler processes such as behavioral and physiological contagion (Edgar & Nicol, 2018) and the reported link between scratching and emotional arousal may suggest that the observed contagious effect of scratching in this study is a behavioral manifestation of emotional contagion.

If scratching is indeed an expression of emotional arousal, then this behavior could serve as a social cue for others (Laidre & Johnstone, 2013). Some other studies have reported on the potential signaling function of scratching. For instance, recent studies show that scratching can be used as a signal to coordinate joint travel, for example, between a mother and infant (Fröhlich, Lee, Setia, Schuppli, & Van Schaik, 2019; Fröhlich, Wittig, & Pika, 2016; Hobaiter & Byrne, 2014), and may be used to initiate grooming (Hobaiter & Byrne, 2014). Another possible communicative function of scratching is to signal social distress, which in turn reduces the likelihood of receiving aggression (Whitehouse et al., 2017). In our study, it is possible that orangutans use scratching in others as a marker of arousal and that the automatic contagion of such information from weakly bonded individuals during tension has an adaptive value.

There was no difference in the probability of scratch contagion between contexts and relationship quality when the orangutan had not seen the triggering scratch, and hence only had auditory cues of this behavior. This can be explained by the fact that the observer had no information about the identity of the initial scratcher which may further highlight a possible link between contagious scratching and a social function.

If scratching indeed serves as a social signal (Fröhlich, Lee et al., 2019; Fröhlich, Wittig et al., 2016; Hobaiter & Byrne, 2014), it is likely intended to change the behavior of the observer with the ultimate goal to benefit the expresser (Bradshaw, 1993; Laidre & Johnstone, 2013). A similar function of scratching is observed during agonistic interactions, where scratching rhesus macaques are less likely to receive aggression (Whitehouse et al., 2017). Because stressed individuals often behave unpredictably (McEwen & Sapolsky, 1995) they can become a potential social stressor (Aureli et al., 1992), especially when they are nonfriends or nonkin (Whitehouse et al., 2017). Hence, increased awareness of such individuals through behavioral contagion may be beneficial for observers and adaptive within group dynamics. Although such adaptive function of behavioral contagion warrants further investigation, we speculate that orangutans can benefit from increased scratch contagion, and potentially contagion of arousal, of weakly bonded individuals during tense contexts, as it may help individuals to prepare for potential unpredictable behaviors of the expresser. This way, scratch contagion becomes adaptive for both the expresser and observer by increasing social cohesion through reducing possible aggression (Rauchbauer, Majdand, Stieger, & Lamm, 2016). While we could not test such aggression‐reducing hypothesis of scratch contagion, this would be interesting to explore in more detail.

It is important to recognize that increased scratch rates have often been observed during tense situations in general, independent of the identity of the individual providing the triggering scratch (Castles & Whiten, 1998; Kaburu et al., 2012; Palagi & Norscia, 2011; Peignot et al., 2004), although there are a number of studies that actually do not find increased scratch rates during anxiety‐provoking circumstances (Aureli & de Waal, 1997; Duboscq, Agil, Engelhardt, & Thierry, 2014; Judge, Griffaton, & Fincke, 2006; Pearson, Reeder, & Judge, 2015). Hence, it is essential to rule out that the heightened scratch contagion between weakly bonded individuals during tense contexts is not merely a by‐product of increased arousal during these contexts. If this were the case, we would expect increased chances of scratch contagion during tense contexts regardless of the relationship quality and whether the triggering scratch was seen or not. This was not the case (see Figure 2). As such, it seems unlikely that the increased contagion observed in our study is a by‐product of higher scratch rates induced by tension, but that it is truly an effect of the context and the relationship quality between the expresser and observer.

In conclusion, this study is the first to provide evidence for the presence of scratch contagion in the orangutan, possibly suggesting a case of emotional contagion. We show that scratch contagion is stronger between weakly bonded individuals when there is tension, demonstrating that it has a possible social function. Our results are relevant for future research on behavioral contagion and emotional contagion as they highlight that contagion is not simply stronger between individuals with a high relationship quality, as is commonly suggested. Furthermore, the variety of contexts in which scratching is observed throughout the literature highlight the complexity of this behavior and the mechanism underlying its contagious effect. Importantly, the degree of scratch contagion may depend on the interaction between contextual factors and social relationships.

## CONFLICTS OF INTEREST

The authors declare that there are no conflicts of interest.

## Supporting information

Supporting information

## Data Availability

The data that support the findings of this study are available from the corresponding author upon reasonable request.
