# Supplementary material for: Low relationship quality predicts scratch contagion during tense situations in orangutans (Pongo pygmaeus)
Source: Am J Primatol. 2020 Apr 24;82(7):e23138. doi: 10.1002/ajp.23138 (PMC7379188; doi:10.1002/ajp.23138)
Supplement: Supplementary file 1 — Supporting information [file AJP-82-e23138-s001.docx]

**Electronic supplementary material**

**Low relationship quality predicts scratch contagion during tense situations in orangutans (*Pongo pygmaeus*)**

Daan W. Laméris^1,2,§^, Evy van Berlo^1^, Elisabeth H.M. Sterck^2,3^, Thomas Bionda^4^, Mariska E. Kret^1,5*^

^1^ Cognitive Psychology Unit, Leiden University, 2333 AK, Leiden, the Netherlands

^2^ Animal Ecology Research Group, Utrecht University, 3584 CH, Utrecht, the Netherlands

^3^ Animal Science Department, Biomedical Primate Research Centre, 2288 GJ, Rijswijk, the Netherlands

^4^ Apenheul Primate Park, 7313 HK, Apeldoorn, the Netherlands

^5^ Leiden Institute for Brain and Cognition, 2300 RC, Leiden, the Netherlands

^§^ Behavioral Ecology and Ecophysiology Group, University of Antwerp, 2610, Antwerp, Belgium

* Correspondence: dr. M.E. Kret; m.e.kret@fsw.leidenuniv.nl

**Supplemental Item – Methods**

| Table S1: Additional information about the study subjects | | | |  |
| --- | --- | --- | --- | --- |
| Name | Gender | Birth year (age) | Minutes of observation | Comments |
| Amos | Male | 2000 (17) | 1120 |  |
| Baju | Male | 2015 (1) | NA | Son of Wattana |
| Binti | Female | 2000 (17) | 1090 |  |
| Dayang | Female | 2005 (12) | 1120 | Adopted by Sandy |
| Kawan | Male | 2010 (7) | 1090 | Son of Wattana |
| Kevin | Male | 1982 (35) | 1110 |  |
| Samboja | Female | 2005 (12) | 1080 | Daughter of Sandy |
| Sandy | Female | 1982 (35) | 1110 |  |
| Silvia | Female | 1965 (52) | 1080 |  |
| Wattana | Female | 1995 (22) | 1110 |  |

| **Ethogram Orangutans (*Pongo pygmaeus*)** | | | |
| --- | --- | --- | --- |
|  | **Behaviour** | **Code** | **Description** |
| Infant | Nursing | N | Infant suckling from nipple unaided (dur) |
|  | Retrieve | R | Retrieve infant from another animal or physical structure (dur) |
| Socio-positive | Allogrooming | GR | One individual picking, stroking or parting of hair over any part of the body with mouth or hands of another individual (dur) |
|  | Being groomed | BG | Focal animal is groomed by another animal (dur) |
|  | Contact | C | Mouth-mouth contact, olfactory inspection, touch (dur) |
|  | Contact sitting | CS | Sitting next to other individual (dur) |
|  | Play | P | Mouth fighting or wrestling (dur) |
| Agonistic | Chase other | CH | Individual pursuing another using any form of locomotion (dur) |
|  | Direct aggression | FI | Biting/hitting/grabbing (dur) |
|  | Display | DI | Charge, shaking of climbing structure/rope (dur) |
|  | Make way | MW | Move out of the way when another animal is approaching (pt) |
| Sexual | Genital contact | GC | One individual touching hand/mouth to another’s genital area (dur) |
|  | Mount | M | Mounting another animal in a copulatory position; genital-genital contact established (dur) |
| Food associated | Drinking | DR | Drink from drink-nipple (pt) |
|  | Feeding | FE | Actively eating, reaching for food, processing or preparing food items (dur) |
|  | Foraging | FO | Searching for food (dur) |
|  | Give food | GF | Give food to another animal (pt) |
|  | Take food | TF | Take food from another animal’s mouth or hand (pt) |
| Locomotion | Brachiate/climbing | CL | Hand-over-hand locomotion (dur) |
|  | Walk | W | Forward or backward locomotion either quadrupedally or bipedally (dur) |
| Facial expression | Funnel face | FF | Maximal pursing of the lips (pt) |
|  | Grimace | GR | Teeth showing, mouth slightly open, corners pulled back (pt) |
| Vocalisation | Grunt | G | Deep, belch-like vocalisation (dur) |
|  | Kiss squeak | KS | Vocalisation made by the intake of air through extended lips (dur) |
|  | Long call | LC | Deep, rumbling vocalisation (dur) |
| Other | Auto-groom | AG | Picking, stroking or parting of own hair over any part of the body (dur) |
|  | Auto-play | AP | Animal plays with food/items alone (dur) |
|  | Caretaker | CA | Interacting/waiting for interactions with the caretaker (dur) |
|  | Nest building | NB | Preparation of day/night nest (dur) |
|  | Object manipulation | OM | Manipulating object with hand/mouth (dur) |
|  | Out of sight | OS | Animal is out of sight (dur) |
|  | Resting | R | Laying (in a nest), not sleeping (eyes opened) (dur) |
|  | Scratching | SC | Auto-scratch (dur) |
|  | Sleeping | SL | Resting without locomotion, eyes are closed (dur) |
|  | Yawn | Y | Opening of mouth and lips with teeth bared or not visible (pt) |

Table S2: Ethogram

Figure S1

**
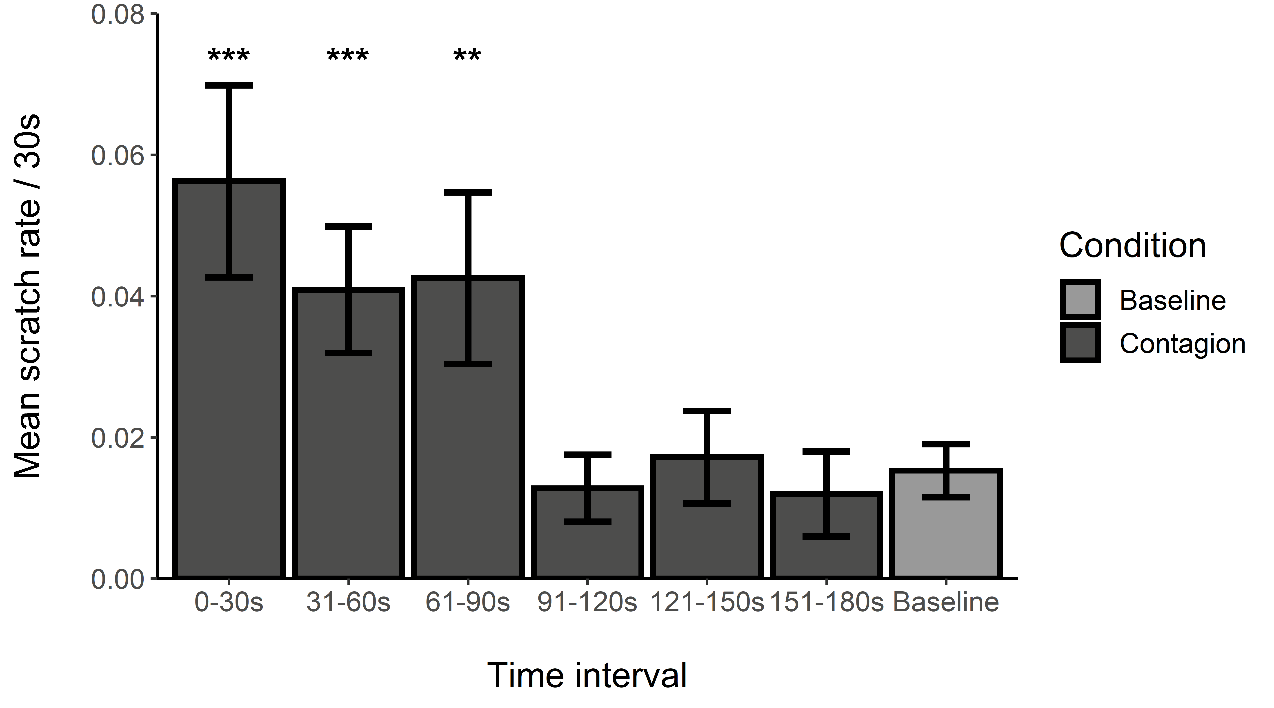
**

Figure S1: Mean scratch rates (±SEM) in the six 30 second intervals in the contagious condition compared to the baseline scratch rate. *** *P* < 0.001, ** *P* < 0.01
